# Computed tomographic signs of hyoid apparatus disease in 165 horses

**DOI:** 10.3389/fvets.2025.1631185

**Published:** 2025-09-19

**Authors:** Bettina Hartl, Carina Strohmayer, Yasamin Vali, Manolis Lyrakis, Sibylle M. Kneissl

**Affiliations:** ^1^Department of Biological Sciences and Pathobiology, Institute of Morphology, Vetmeduni, Austria; ^2^Diagnostic Imaging, Clinical Department of Small Animals and Horses, Vetmeduni, Austria; ^3^Platform for Bioinformatics and Biostatistics, Department of Biological Sciences and Pathobiology, Vetmeduni, Austria

**Keywords:** hyoid bone disease, computed tomography, temporohyoid joint, equine, hyoid apparatus, degenerative change, styloceratohyoid/epiceratohyoid joint, riding problems

## Abstract

**Introduction:**

Horses with hyoid bone disease may present with clinical symptoms such as exercise intolerance, resistance to ridden work, anorexia, or headshaking. This study aims to describe the computed tomographic (CT) signs and prevalence of hyoid disease and to evaluate their association with other CT changes of the head.

**Methods:**

In this study, four investigators conducted a retrospective analysis of their findings after reviewing the medical records of 200 horses that had undergone CT scans between 2009 and 2019. A total of 32 parameters were analyzed, and associations were evaluated between those parameters and the effects of age, weight, and use on the development of hyoid bone disease.

**Results:**

The temporohyoid joint (THJ) exhibited the most common CT signs, with 52% of cases showing marginal exostosis of the temporal bone, 44.4% displaying osteophytes of the proximal stylohyoid, and 40.2% demonstrating reduced tympanohyoid on the examined sides. The most frequently observed CT signs at the styloceratohyoid/epiceratohyoid joint were marginal exostosis of the distal stylohyoid, identified in 29.0% of the examined sides, and osteoproliferative changes of the proximal ceratohyoid, present in 16.5% of the examined sides. CT signs of disease were rarely found at the ceratobasihyoid joint. Pathological CT signs of the ceratobasihyoid joint were associated with those of the styloceratohyoid/epiceratohyoid joint, and disease in this joint was associated with those of the THJ. The findings of the present study revealed an age-related effect on the occurrence of marginal exostosis and osseous cyst-like lesions (OCLLs) of the temporal bone, as well as on OCLLs and osteophytes of the proximal stylohyoid, and on the thickening of the THJ, and a reduction in the tympanohyoid. Age was also found to influence the incidence of the two styloceratohyoid/epiceratohyoid joint CT signs: marginal exostosis of the distal stylohyoid and osteoproliferative changes at the proximal ceratohyoid. The weight and use of the horses did not affect the occurrence of the evaluated changes.

**Conclusion:**

CT signs of disease of the hyoid apparatus are common and should be considered as part of the differential diagnosis when examining a horse with poor performance, riding complaints, or headshaking.

## 1 Introduction

The equine hyoid apparatus stabilizes the tongue, pharynx, and larynx. Disease of the hyoid bone can be manifested by clinical symptoms such as exercise intolerance, difficulty riding, headshaking, or anorexia (1–4). It is therefore important to raise awareness that hyoid bone diseases should be considered as a potential factor when these clinical symptoms arise.

The equine hyoid apparatus consists of the paired tympanohyoid, stylohyoid, epihyoid, ceratohyoid, and the unpaired basihyoid with the paired thyrohyoid processes. The tympanohyoid attaches the stylohyoid to the skull, the basihyoid is embedded in the tongue, and the thyrohyoid process articulates with the thyroid cartilage of the larynx. The various bones of the hyoid are connected via synovial joints, except for the temporohyoid joint (THJ), where the stylohyoid is connected to the temporal bone (TB) via the tympanohyoid, which consists of hyaline cartilage (5). The styloceratohyoid/epiceratohyoid joint (STCJ) connects either the stylohyoid, in case the epihyoid is fused with the stylohyoid, or the epihyoid with the ceratohyoid. The ceratobasihyoid joint (CBJ) is a connection between the ceratohyoid and the horizontal part of the basihyoid.

Imaging techniques used to assess the hyoid apparatus include radiography (2–4, 6), ultrasonography (4), endoscopy (1–4, 6), magnetic resonance (MR) imaging (6), and computed tomography (CT) (1–4, 6–9). In 20/24 horses, radiographs revealed bony changes of the THJ, tympanic bulla, or stylohyoid in horses with temporohyoid osteoarthropathy (THO) (6). In one case of hyoid malformation, a caudal deviation of the stylohyoid was visible (2). In both of these cases, the definitive diagnosis was achieved using CT. However, head radiographs were interpreted as normal in a case of lingual process (LP) fracture (3), and no bony trauma was detected in a case of basihyoid fracture (4). In both of these cases, the definitive diagnosis was achieved using CT. Ultrasonography can be used to assess the ventral aspects of the hyoid bone and was employed in a previous study to monitor a basihyoid fracture, for which the initial diagnosis was performed using CT (1, 4). Guttural pouch endoscopy offers a good overview of the THJ and the proximal stylohyoid, but the middle ear and the ventral part of the hyoid are not accessible (7, 10). MR imaging offers better visualization of soft tissues, enabling the identification of inflammatory fluid accumulation in the middle and inner ear; however, it is less accurate in assessing osseous structures (6), and general anesthesia is required for head MRs. CT is currently the most valuable imaging modality for evaluating the hyoid bone (8, 11, 12), as it can reveal abnormalities that cannot be detected with radiography (3, 4, 7) or endoscopy (1–4, 7). Furthermore, CT of the equine head can be performed standing, and recovering a neurologic horse from anesthesia presents an increased risk to the patient (13), a concern particularly relevant as neurological symptoms are frequently observed in horses with THO (6, 14).

Normal CT anatomy of the entire hyoid apparatus was recently described (5). Several CT signs have been described in the literature, but these are almost exclusively confined to the THO (6, 7, 9, 14, 15), with a few exceptions (16–18). CT signs of THO include: sclerosis (9), marginal exostosis and fracture of the TB (15), thickening (7), bridging and fusion of the THJ (15), as well as osteophytes at the proximal stylohyoid (18), and bony proliferations at the STCJ joint (7). Other published abnormalities of the hyoid apparatus include THJ subluxation (19), and stylohyoid (1, 20) or basihyoid fracture (4). Malformation of the hyoid apparatus (2, 21), arthropathy (17) of the STCJ, and LP fracture (3) have also been described.

The objective of this explorative study was: to (i) assess the frequency of pathological CT signs in individual articulations of the hyoid apparatus; (ii) to characterize the type of CT signs for each joint; (iii) to evaluate the effect of age, use, and weight on the occurrence and type of CT signs which appear, and (iv) to analyze the probability of concurrent occurrence of defined pathological CT signs in the hyoid apparatus, temporomandibular joint, and cheek teeth.

## 2 Materials and methods

### 2.1 Medical record review

Medical records of 200 horses that underwent a CT scan of the head between 2009 and 2019 were retrieved from the archive of diagnostic Imaging at the Clinical Department for Companion Animals and Horses, University of Veterinary Medicine, Vienna, Austria. Information obtained from the medical record included age, breed, sex, use, and indication for the CT scan of the head. For horses that had undergone multiple CT scans, only the first CT scan was included in the study. CT scans were excluded if the entire hyoid apparatus was not adequately visualized. Also excluded were the CT scans of horses with developmental stages of the hyoid apparatus corresponding to less than 3 years of age, as described by Hartl et al. (5), and postmortem CT scans were excluded from the assessment of signs of disease of the external and/or middle ear.

### 2.2 Computed tomography analysis

Of the 200 horses analyzed, the first 15 horses on the list, sorted by the date of the CT scan and meeting the inclusion criteria, were used as the pilot study. Each investigator (BH, CS, YV, and SMK) met in a total of six online meetings for this pilot study to discuss the analyzed differences and agree upon a scoring methodology. The four investigators were an equine veterinarian and anatomist (BH), a senior experienced radiologist (SMK), a European College of Veterinary Diagnostic Imaging (ECVDI) diplomate/board-certified veterinary radiologist (CS), and a third-year resident in ECVDI/radiology resident (YV). For the pilot study, investigators met online twice a week at the start of the study to discuss requirements and resolve scoring discrepancies. After the first month, meetings were held every 3–4 weeks to address questions. Following the initial 15-horse pilot study, the procedure was modified: two examiners (SMK and either CS or YV) scored images, one examiner (BH) identified differences, and a fourth examiner (CS or YV, depending on the initial scoring) resolved discrepancies. If the fourth examiner disagreed, an online meeting was held to review images and reach a consensus. Weekly work orders and CT datasets were shared via the Nextcloud platform (version 28.0.2, Nextcloud GmbH, Stuttgart, Germany).

Head CT scans reported in this study were acquired using either a 128-slice scanner (Somatom X.cite, Siemens Healthineers, Erlangen, Germany) or, for cases prior to June 2021, a 16-slice scanner (Somatom Emotion 16, Siemens Healthineers, Erlangen, Germany). Imaging parameters included a tube voltage of 130 kVp, a tube current–time product of 300 mAs, a pitch of 0.3–1.5, a reconstructed slice thickness of 0.75–1.5 mm, and a rotation time of 0.3–1 s. The field of view was either 35 or 50 cm, and the matrix was 512 × 512. Patients were positioned in dorsal recumbency, with the specimen in sternal recumbency, and images were acquired in the transverse plane using reconstruction algorithms for both bone and soft tissue.

### 2.3 Scoring of CT signs

The principal investigator (BH) compiled a list of hyoid CT signs of disease published in the literature and discussed it with the other examiners (SMK, CS, and YV). This compilation has been adjusted to reflect any diseases discussed in the online sessions. Before the reviewers began evaluating the CT scans, a CT scan showing no diseases in the hyoid apparatus was jointly reviewed. Images were reviewed on DICOM workstations (Jive, Visus, Bochum, Germany). All reviewers assessed CT scans in all three planes using multiplanar reconstruction (MPR) in the bone window, separately for the left and right sides. The gold standard for CT image interpretation was the transverse plane with unchanged slice thickness. Maximum intensity projection (MIP) and 5-mm multiplanar reconstructions were used in cases where there were minimal changes or uncertainty in interpretation.

The CT signs of hyoid apparatus diseases were assessed as present (1) or absent (0) with no additional grading. The CT signs of concurrent head diseases were assessed as present (1) or absent (0), also with no additional grading. All assessed CT signs are listed in Table 1.

**Table 1:** List of assessed CT-signs for hyoid apparatus diseases and concurrent head diseases.

Assessed CT signs for hyoid apparatus diseases	Assessed concurrent head diseases
TB: External/middle ear disease	Mandibular cheek teeth disease
TB: Fracture	Maxillary cheek teeth disease
TB: Osteopenia	Perihyoid change
TB: Sclerosis	TMJ disease
THJ: Marginal exostosis TB	
THJ: OCLL TB	
THJ: Ankylosis	
THJ: Bridging	
THJ: Reduced tympanohyoid	
THJ: Subluxation	
THJ: Thickening	
THJ: OCLL proximal stylohyoid	
THJ: Osteophytes proximal stylohyoid	
THJ: Remodeling proximal stylohyoid	
ST: Fracture	
STCJ: Marginal exostosis distal stylohyoid	
STCJ: OCLL distal stylohyoid	
STCJ: Separate epihyoid	
STCJ: Bridging	
STCJ: OCLL proximal ceratohyoid	
STCJ: Osteoproliferative changes proximal ceratohyoid	
CBJ: Marginal exostosis distal ceratohyoid	
CBJ: OCLL distal ceratohyoid	
CBJ: Ankylosis	
CBJ: Marginal exostosis basihyoid	
CBJ: OCLL basihyoid	
LP: Asymmetry	
LP: Fracture	

CBJ, Ceratobasihyoid joint; LP, Lingual process; OCLL, Osseous cyst-like lesion; ST, Stylohyoid; STCJ, Styloceratohyoid/epiceratohyoid joint; TB, Temporal bone; THJ, Temporohyoid joint; TMJ, Temporomandibular joint.

#### 2.3.1 Assessment of hyoid disease

##### 2.3.1.1 Temporal bone disease

###### 2.3.1.1.1 External/middle ear disease

The presence of a homogenous soft tissue opacity within the tympanic bulla, a hyperattenuating, deformed, or thickened wall of the tympanic bulla, loss of visible volume of the external acustic meatus, or soft tissue opacity within the canal was recorded as “present” (score = 1) for external/middle ear disease.

###### 2.3.1.1.2 Fracture of the temporal bone

A TB fracture was defined as a hypoattenuating line traversing the bone, consistent with type 1 or type 2 fractures as described by Tanner et al. (15). TB fracture visible in at least two planes was classified as present (score = 1).

###### 2.3.1.1.3 Osteopenia of the temporal bone

Osteopenia of the temporal bone was confirmed based on the observation of increased radiolucency in the trabecular bone of the caudodorsal part of the petrous temporal bone. Osteopenia visible in at least two planes was classified as present (score = 1).

###### 2.3.1.1.4 Sclerosis of the temporal bone

Sclerosis of the TB was confirmed based on the observation of increased bone density (following the CT characteristics of TB sclerosis illustrated in a study by Rullan-Majol et al. (9)). Sclerosis visible in at least two planes was classified as present (score = 1).

##### 2.3.1.2 Temporohyoid joint disease

###### 2.3.1.2.1 Marginal exostosis of the temporal bone

Marginal exostosis (ME) of the TB was confirmed when mild periarticular osteoproliferation occurred at the lateral, medial, rostral, or caudal margin of the TB (following grade 1 scoring demonstrated in a study by Aleman et al. (14)). ME was noted as an osteoproliferation extending ventrally beyond the level of the styloid process on the lateral side and ventrally beyond the ventral border of the tympanic bulla on the medial side. An osteoproliferation on the tympanic bulla wall involving the medial part of the THJ was not considered as ME and classified as absent (0). ME visible in at least two planes was classified as present (score = 1).

###### 2.3.1.2.2 Osseous cyst-like lesion of the temporal bone

An osseous cyst-like lesion (OCLL) of the TB was confirmed when a well-defined radiolucent area was identified adjacent to the tympanohyoid [following histologically proven OCLL in a separate study (unpublished data)]. An OCLL visible in at least two planes was classified as present (score = 1).

###### 2.3.1.2.3 Ankylosis of the temporohyoid joint

Ankylosis of the THJ was confirmed when severe periarticular osteoproliferation occurred between the TB and the stylohyoid (following grade 3 scoring discussed in the study by Tanner et al. (15)). Ankylosis was noted when no gap between the TB and the stylohyoid and complete fusion of the THJ were visible. Ankylosis visible in at least two planes was classified as present (score = 1).

###### 2.3.1.2.4 Bridging of the temporohyoid joint

Bridging of the THJ was confirmed when moderate periarticular osteoproliferation was observed between the TB and the stylohyoid, as demonstrated in a study by Tanner et al. (following grade 2 and 3 scoring) (15) was identified. Bridging was noted when a gap between the TB and the stylohyoid was visible. Bridging visible in at least two planes was classified as present (score = 1).

###### 2.3.1.2.5 Reduced tympanohyoid

Reduced tympanohyoid was confirmed when narrowing of the tympanohyoid occurred (following the illustration by Dixon et al. (2)) who showed subtle narrowing and Hilton et al. (7) showed severe narrowing. Reduced tympanohyoid visible in at least two planes was classified as present (score = 1).

###### 2.3.1.2.6 Subluxation of the temporohyoid joint

Subluxation of the THJ was confirmed when a subluxation, displacement of the stylohyoid, and/or soft tissue swelling was observed (following the example image of Manso-Diaz et al. (19)). Subluxation visible in at least two planes was classified as present (score = 1).

###### 2.3.1.2.7 Thickening of the temporohyoid joint

Thickening of the THJ was recorded when it occurred (following the study by Hilton et al. (7) who demonstrated a mild, moderate, or severe thickening of the THJ). Thickening of the THJ visible in at least two planes was classified as present (score = 1).

###### 2.3.1.2.8 Osseous cyst-like lesion of the proximal stylohyoid

OCLL of the proximal stylohyoid was confirmed when a well-defined radiolucent area was identified adjacent to the tympanohyoid. OCLL visible in at least two planes was classified as present (score = 1).

###### 2.3.1.2.9 Osteophytes of the proximal stylohyoid

Osteophytes of the proximal stylohyoid bone were confirmed when osteophytes at the marginal aspect of the stylohyoid bone were observed extending into the tympanohyoid cartilage, as described by Naylor et al. (18). Osteophytes were classified as present (score = 1) if they were visible in at least two imaging planes.

###### 2.3.1.2.10 Remodeling of the proximal stylohyoid

Remodeling of the stylohyoid was confirmed when osteoproliferation and/or osteolysis occurred in the body of the stylohyoid; however, not at the articular surface. Remodeling visible in at least two planes was classified as present (score = 1).

##### 2.3.1.3 Stylohyoid disease

###### 2.3.1.3.1 Fracture of the stylohyoid

A stylohyoid fracture was confirmed when a hypoattenuating line traversing the bone, with or without displacement, was identified. Fractures were classified as present (score = 1) if they were visible in at least two imaging planes.

##### 2.3.1.4 Styloceratohyoid/epiceratohyoid joint disease

###### 2.3.1.4.1 Marginal exostosis of the distal stylohyoid

The ME of the stylohyoid was confirmed when periarticular osteoproliferation was observed at its distal articular end. The ME of the distal stylohyoid was classified as present (score = 1) if it was visible in at least two imaging planes.

###### 2.3.1.4.2 Osseous cyst-like lesion of the distal stylohyoid

OCLL of the distal stylohyoid was confirmed when a well-defined radiolucent area was identified adjacent to the articular surface of the STCJ. OCLL visible in at least two planes was classified as present (score = 1).

###### 2.3.1.4.3 Separate epihyoid

The presence (score = 1) or absence (score = 0) of a separate epihyoid was recorded. According to Hartl et al. (5), a separate epihyoid is most effectively visualized on sagittal thick-slice MPRs or in a dorsal plane.

###### 2.3.1.4.4 Bridging of the styloceratohyoid/epiceratohyoid joint

Bridging of the styloceratohyoid joint was recorded when the stylohyoid and epihyoid were fused, and of the epiceratohyoid joint when the epihyoid was not fused to the stylohyoid. The fusion of the epiceratohyoid joint was also attributed to the bridging of the joint. Bridging visible in at least two planes was classified as present (score = 1).

###### 2.3.1.4.5 Osseous cyst-like lesion of the proximal ceratohyoid

OCLL of the proximal ceratohyoid was confirmed when a well-defined radiolucent area was identified adjacent to the articular surface of the STCJ [following histologically proven OCLL in a separate study (unpublished data)]. OCLL visible in at least two planes was classified as present (score = 1).

###### 2.3.1.4.6 Osteoproliferative change of the proximal ceratohyoid

Osteoproliferative changes of the proximal ceratohyoid were confirmed when periarticular osteoproliferation was observed at its proximal aspect, consistent with the CT appearance of osseous proliferation of the ceratohyoid bone described by Hilton et al. (7). These changes were classified as present (score = 1) if they were visible in at least two imaging planes.

##### 2.3.1.5 Ceratobasihyoid joint disease

###### 2.3.1.5.1 Marginal exostosis of the distal ceratohyoid

The ME of the ceratohyoid was confirmed when periarticular osteoproliferation was observed at its distal articular end. The ME of the distal ceratohyoid was classified as present (score = 1) if it was visible in at least two imaging planes.

###### 2.3.1.5.2 Osseous cyst-like lesion of the distal ceratohyoid

OCLL of the distal ceratohyoid was confirmed when a well-defined radiolucent area was identified adjacent to the articular surface of the CBJ. OCLL visible in at least two planes was classified as present (score = 1).

###### 2.3.1.5.3 Ankylosis of the ceratobasihyoid joint

Ankylosis of the CBJ was confirmed when no gap between the ceratohyoid and basihyoid bones was observed, along with evidence of complete joint fusion. Ankylosis was classified as present (score = 1) if it was visible in at least two imaging planes.

###### 2.3.1.5.4 Marginal exostosis of the basihyoid

ME of the basihyoid was confirmed when periarticular osteoproliferation was observed at its horizontal part at the articular end. The ME of the basihyoid was classified as present (score = 1) if it was visible in at least two imaging planes.

###### 2.3.1.5.5 Osseous cyst-like lesion of the basihyoid

OCLL of the distal ceratohyoid was confirmed when a well-defined radiolucent area was identified adjacent to the articular surface of the CBJ. OCLL visible in at least two planes was classified as present (score = 1).

##### 2.3.1.6 Lingual process disease

###### 2.3.1.6.1 Asymmetry of the lingual process

Asymmetry of the LP was confirmed when its longitudinal axis did not align with the longitudinal axis of the head. Asymmetry visible in at least two planes was classified as present (score = 1).

###### 2.3.1.6.2 Fracture of the lingual process

An LP fracture was recorded when a discontinuity of the bone, bone fragments near the fracture site, misalignment, or soft tissue swelling around the fracture site was present. A fracture visible in at least two planes was classified as present (score = 1).

#### 2.3.2 Assessment of concurrent head diseases

##### 2.3.2.1 Perihyoid change

Perihyoid changes were documented in cases where mineralization of the soft tissues surrounding the larynx or mandibular fractures extending to the level of the hyoid were observed. Changes were classified as present (score = 1) if they were visible in at least two imaging planes.

##### 2.3.2.2 Selected cheek teeth diseases

In this study, the following selected cheek teeth diseases were included: apical tooth root abscess, tooth loss, or a maxillary or mandibular fracture with involvement of an alveolus. All cheek teeth were evaluated.

Apical tooth root abscess was diagnosed based on the presence of a radiolucent area surrounding the tooth apex, increased radiodensity in the bone adjacent to the periapical lucency, or evidence of irregularity, resorption, or enlargement of the affected tooth root. The presence of a selected cheek teeth disease affecting a single tooth within an arcade was classified as present (score = 1) for the respective arcade if it was visible in at least two imaging planes.

##### 2.3.2.3 Temporomandibular joint disease

Temporomandibular joint (TMJ) disease was confirmed when joint asymmetry, flattening, remodeling, OCLL, sclerosis, lysis, or osteoproliferative changes of the mandibular condyle or articular tubercle of the temporal bone, disk derangement/ mineralization, or osteoproliferative changes on the lateral margin of the mandibular condyle, and CT signs based on the images published in a study by Carmalt et al. (22) were observed. TMJ disease was classified as present (score = 1) if it was visible in at least two imaging planes.

### 2.4 Statistical analysis

Statistical analyses were performed in R (R version 4.4.1) (23). Only a subset of CT-parameters (22), with sufficient numbers to qualify as a disease, was used for association tests, and even fewer (13) were used for the evaluation of effects (Supplementary Table S1). For all results, significance was declared at 5% cut-off.

Pairwise association tests between CT parameters were evaluated using Cochran–Mantel–Haenszel (CMH) tests, which accounted for the side of observation (left, right) using the function mantelhaen.test with default settings. If a zero was present in the contingency tables of both sides for a pair of parameters, one was added in all the cells of both tables. Multiple association tests were corrected for multiple testing using the Bonferroni–Holm method (function p. adjust). Results from the association tests were visualized in heatmaps (package pheatmap, version 1.0.12, function pheatmap) (24).

Effects on observed diseases for each parameter were evaluated via binary logistic regression models (function glm, option “family = binomial (link = ‘logit’)”). For most CT parameters, only a few horses exhibited different observations between sides. Thus, data were aggregated (one observation per horse per parameter) and CT signs were coded as “0” (no disease) and “1” (at least one disease per horse) (Supplementary Table S1). In all models, the CT sign was fitted as a binary response, and the age (in years), weight (in one-tenth of a metric ton), and usage (up to seven levels; a few horses were excluded, if necessary, to avoid complete separation regarding usage, see Supplementary Table S1) were fitted as fixed effects. Breed was not fitted as a fixed effect, due to multicollinearity with other fixed effects (multicollinearity between fixed effects was evaluated using variance–inflation factors, package car, version 3.1.3, function vif (25)). The overall significance of usage was evaluated using a likelihood ratio test with ANOVA function, comparing the full model to a reduced model without usage as a fixed effect. For each fixed effect (age, weight, and overall significance of usage), results from all models were aggregated and corrected for multiple testing via the Bonferroni–Holm method (function p. adjust).

## 3 Results

### 3.1 Study population

Of the 200 horses selected for this study, 165 horses met the inclusion criteria. The study population consisted of 11 Stallions, 68 Mares, 78 Geldings, and 8 horses with unknown sex. Age ranged from 3 to 29 years (median age: 13 years). The majority of the horses (66) were used as leisure horses, followed by dressage horses (24), show jumpers (12), young horses (7), breeding horses (6), and 20 horses used for other purposes. In 30 horses, no records of use were available. The weight ranged from 65 to 720 kg, with a median weight of 503 kg; however, the weights of 10 horses were not recorded. The majority of the horses were Warmbloods (90), followed by small horses (23). The most common indications for a CT scan were a skull mass (63), sinusitis (41), and a jaw fracture (27).

### 3.2 CT-evaluation

**Figure 1:**
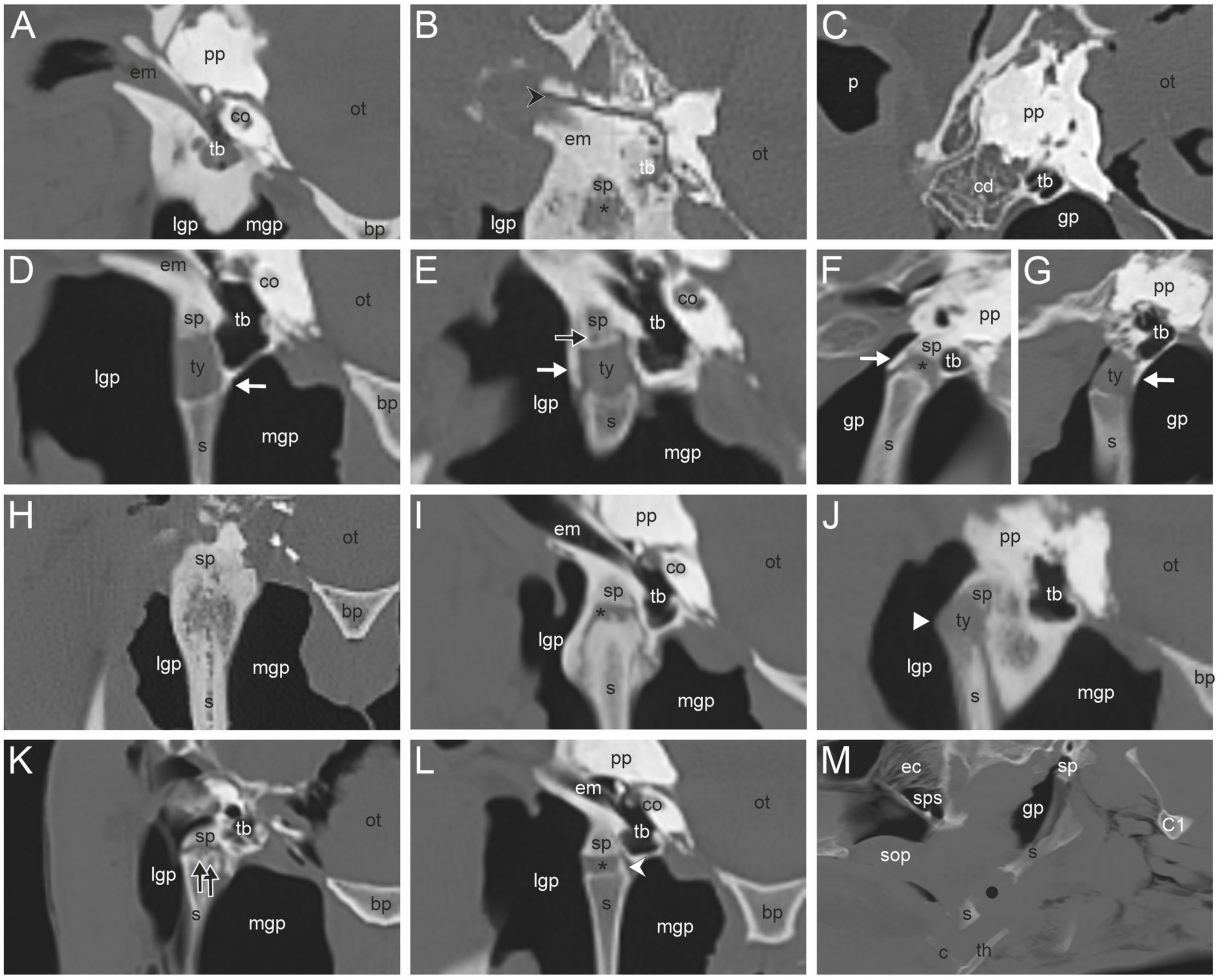
**(A)** External/middle ear disease associated with sclerosis of the temporal bone; **(B)** Fracture of the temporal bone (black directional arrowhead); **(C)** Osteopenia in the caudodorsal portion of the petrous temporal bone (cd). Marginal exostoses of the temporal bone at the medial **(D)**, lateral **(E)**, rostral **(F)** and, caudal **(G)** side (white arrow); **(E)** Osseous cyst-like lesion (OCLL) of the temporal bone (black arrow); **(H)** Ankylosis of the temporohyoid joint (THJ) and remodeling of the proximal stylohyoid; **(I)** Bridging and thickening of the THJ; **(B,F,I,K,L)** Reduced tympanohyoid (asterisk); **(J)** Subluxation of the THJ (white arrowhead); **(K)** OCLL of the proximal stylohyoid; **(L)** Osteophyte proximal stylohyoid (white directional arrowhead); **(M)** Fracture of the stylohyoid (black dot). Transverse CT images in a bone window of the area around the temporohyoid joint of a horse without records **(A)**, a 22-year-old Warmblood mare **(B,H)**, a Warmblood without gender and age record **(C)**, a 5-year-old Warmblood gelding **(D)**, a 14-year-old Warmblood mare **(E)**, a 26-year-old Standardbred mare **(I)**, an 8-year-old Warmblood mare **(J)**, an 8-year-old Warmblood gelding **(L)**, and a 23-year-old Warmblood mare **(K)**. Sagittal CT images in a bone window of the THJ of a 23-year-old Warmblood mare **(F)** and a 17-year-old Haflinger mare **(G)**. Sagittal CT image of the area around the stylohyoid of a 19-year-old Warmblood mare **(M)**. In image **(F,G,M)** rostral is to the left. In image **(A–E,H–L)** medial is to the right. (bp) basilar part of the occipital bone, (c) ceratohyoid, (co) cochlea, (C1) first cervical bone, (ec) ethmoidal conchae, (em) external acustic meatus, (gp) guttural pouch, (lgp) lateral guttural pouch, (mgp) medial guttural pouch, (ot) osseous tentorium, (p) pinna, (pp) petrosal pyramid, (s) stylohyoid, (sop) soft palate, (sp) styloid process, (sps) sphenopalatine sinus, (th) thyrohyoid, (tb) tympanic bulla, (ty) tympanohyoid.

**Figure 2:**
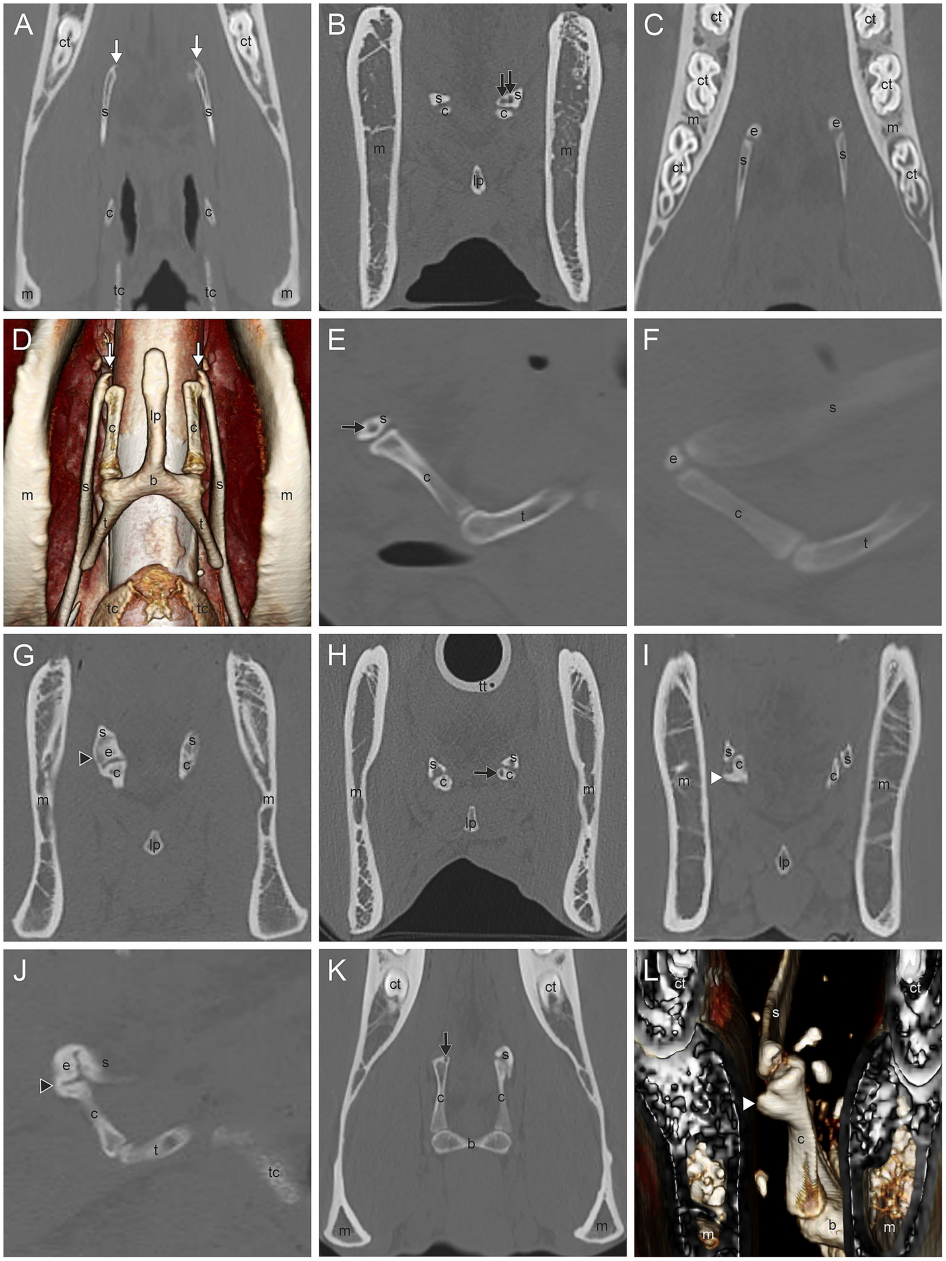
**(A,D)** Marginal exostosis of the distal stylohyoid (white arrow); **(B,E)** Osseous cyst-like lesions (OCLL) of the distal stylohyoid (black arrow); **(C,F)** Separate epihyoid (e); **(G,J)** Bridging of the stylohyoid (s) with the ceratohyoid (c) (black arrowhead); **(H,K)** OCLL of the proximal ceratohyoid (black arrow); **(I,L)** Osteoproliferative changes of the proximal ceratohyoid (white arrowhead). Dorsal CT image of the styloceratohyoid/epiceratohyoid joint of a 11-year-old Warmblood mare **(A)**, a Warmblood without gender and age record **(C)**, and a 16-year-old Warmblood mare **(K)**. Transverse CT images of the same area of a horse without records **(B)**, a Warmblood with no gender and age record, **(G)**, a 24-year-old Warmblood mare **(I)**, and of the same horse as in image **K (J)**. Sagittal CT image of the same horse as in image **G (J),** of the same horse as in image **B (E)**, and of the same horse as in image **C (F)**. 3D surface model of the same horse as in image **I** with a view from rostromedial to caudolateral oblique **(L)** and of the same horse as in image **A** with a ventral view **(D)**. Image **(A–C,E–K)** in bone window. The 3D surface models **(D,L)** were acquired in soft tissue algorithm. (b) basihyoid, (c) ceratohyoid, (ct) cheek tooth, (e) epihyoid, (lp) lingual process, (m) mandible, (s) stylohyoid, (t) thyrohyoid process, (tc) thyroid cartilage, (tt) tracheal tube.

**Figure 3:**
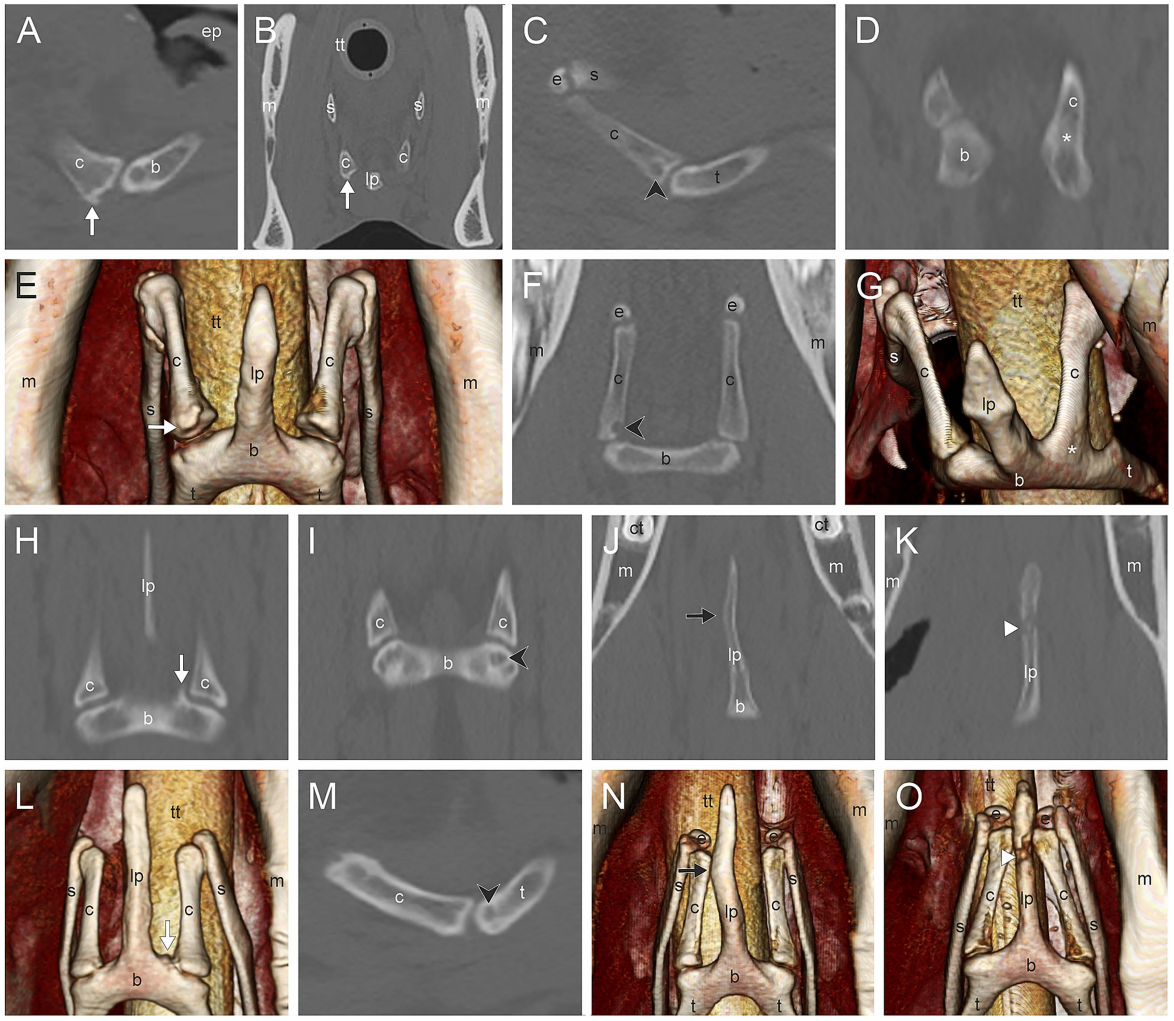
**(A,B,E)** Marginal exostosis of the distal ceratohyoid (c) (white arrow); **(C)**, **(F)** Osseous cyst-like lesions (OCLL) of the distal ceratohyoid (black directional arrowhead); **(D,G)** Ankylosis of the ceratobasihyoid joint (asterisk); **(H,L)** Marginal exostosis of the basihyoid (b) (white arrow); **(I,M)** OCLL of the basihyoid (black directional arrowhead); **(J,N)** Asymmetry of the lingual process (lp) (black arrow); **(K,O)** Fracture of the lingual process (white arrowhead). Sagittal CT image **(A)**, transverse CT image **(B)** and 3D surface model with a ventral view **(E)** of the ceratobasihyoid joint of a 17-year-old Haflinger mare. Sagittal CT image **(C)** and dorsal CT image **(F)** of a Warmblood without gender and age record. Dorsal CT image **(D)** and 3D surface model with a view from craniolateroventral of the ceratobasihyoid joint **(G)** of a Warmblood mare without age record. Dorsal CT image and 3D surface model with a ventral view of the same area in a 11-year-old Warmblood gelding **(H,L)**, a 6-year-old Warmblood mare **(J,N)**, and a 6-year-old Thouroughbred gelding **(K,O)**. Images **(A–D,F,H–K,M)** are displayed in a bone window, images **(E,G,L,N,O)** are 3D surface models. In images **(A,C,M)** left is rostral. In images **(D–F,H–L,N,O)** rostral is at the top. (b) basihyoid, (c) ceratohyoid, (ct) cheek tooth, (e) epihyoid, (ep) epiglottis, (lp) lingual process, (m) mandible, (s) stylohyoid, (t) thyrohyoid process, (tt) tracheal tube.

The most common CT findings were marginal exostoses of the TB (Figures 1D–G) in 52% (166/319), osteophytes of the proximal stylohyoid (Figure 1L) in 44.4% (143/322), a reduced tympanohyoid (Figures 1F,I,K,L) in 40.2% (130/323), and a thickening of the joint (Figures 1B,I,K) in 23.5% (76/323) of the sides examined (Table 2; Supplementary Table S1). Signs of external/middle ear disease (Figure 1A) were found in 2.2% (6/273) of the ears evaluated. In the area of the STCJ, a separate epihyoid (Figures 2C,F) was most common in 38.7% (127/328), marginal exostosis on the distal stylohyoid (Figures 2A,D) in 29% (95/328), osteoproliferative changes on the proximal ceratohyoid (Figures 2I,L) in 16.5% (54/238), followed by OCLL of the proximal ceratohyoid (Figures 2H,K) in 7.6% (25/328), OCLL of the distal stylohyoid (Figures 2B,E) in 7.3% (24/328), and bridging of the joint (Figures 2G,J) in 2.1% (7/328) sides. Diseases of the CBJ were rare, with marginal exostosis of the distal ceratohyoid (Figures 3A,B,E) in 6.4% (21/328), marginal exostosis of the basihyoid (Figures 3H,L) in 2.7% (9/328), OCLL of the basihyoid (Figures 3I,M) in 1.5% (5/328), OCLL of the distal ceratohyoid (Figures 3C,F) in 1.2% (4/328), and ankylosis of the joint (Figures 3D,G) in 0.6% (2/328). Although the 3D reconstructions were not used for evaluation, they are shown in Figures 2, 3 to illustrate the changes more effectively.

**Table 2:** List of assessed CT-imaging findings and their prevalence.

Parameter evaluated	Number of changes per evaluated side/horse (in case of LP)	% of evaluated sides/horses (in case of LP)
TB: External/middle ear disease	6/273	2.2
TB: Fracture	1/293	0.3
TB: Osteopenia	22/256	8.6
TB: Sclerosis	2/256	0.8
THJ: Marginal exostosis TB	166/319	52.0
THJ: OCLL TB	34/313	10.9
THJ: Ankylosis	5/323	1.5
THJ: Bridging	26/323	8.0
THJ: Reduced tympanohyoid	130/323	40.2
THJ: Subluxation	1/323	0.3
THJ: Thickening	76/323	23.5
THJ: OCLL proximal stylohyoid	44/323	13.6
THJ: Osteophytes proximal stylohyoid	143/322	44.4
THJ: Remodeling proximal stylohyoid	13/322	4.0
ST: Fracture	1/322	0.3
STCJ: Marginal exostosis distal stylohyoid	95/328	29.0
STCJ: OCLL distal stylohyoid	24/328	7.3
STCJ: Separate epihyoid	127/328	38.7
STCJ: Bridging	7/328	2.1
STCJ: OCLL proximal ceratohyoid	25/328	7.6
STCJ: Osteoproliferative changes proximal ceratohyoid	54/328	16.5
CBJ: Marginal exostosis distal ceratohyoid	21/328	6.4
CBJ: OCLL distal ceratohyoid	4/328	1.2
CBJ: Ankylosis	2/328	0.6
C-B: Marginal exostosis basihyoid	9/328	2.7
CBJ: OCLL basihyoid	5/328	1.5
LP: Asymmetry	9/161	5.6
LP: Fracture	1/161	0.6
CHD: Perihyoid change	11/326	3.4
CHD: Mandibular cheek teeth disease	39/290	13.4
CHD: Maxillary cheek teeth disease	83/287	28.9
CHD: TMJ disease	60/323	18.6

CBJ, Ceratobasihyoid joint; CHD, Concurrent head diseases; LP, Lingual process; OCLL, Osseous cyst-like lesion; ST, Stylohyoid; STCJ, Styloceratohyoid/epiceratohyoid joint; TB, Temporal bone; THJ, Temporohyoid joint; TMJ, Temporomandibular joint.

LP asymmetry (Figures 3J,N) was noted in 5.6% (9/161) and a fracture (Figures 3K,O) in 0.6% (1/161) of the horses. TMJ diseases occurred in 18.6% (60/323), diseases in the mandibular cheek teeth in 13.4% (39/290), and in the maxillary cheek teeth in 28.9% (83/287).

### 3.3 Associations between the parameters

**Figure 4:**
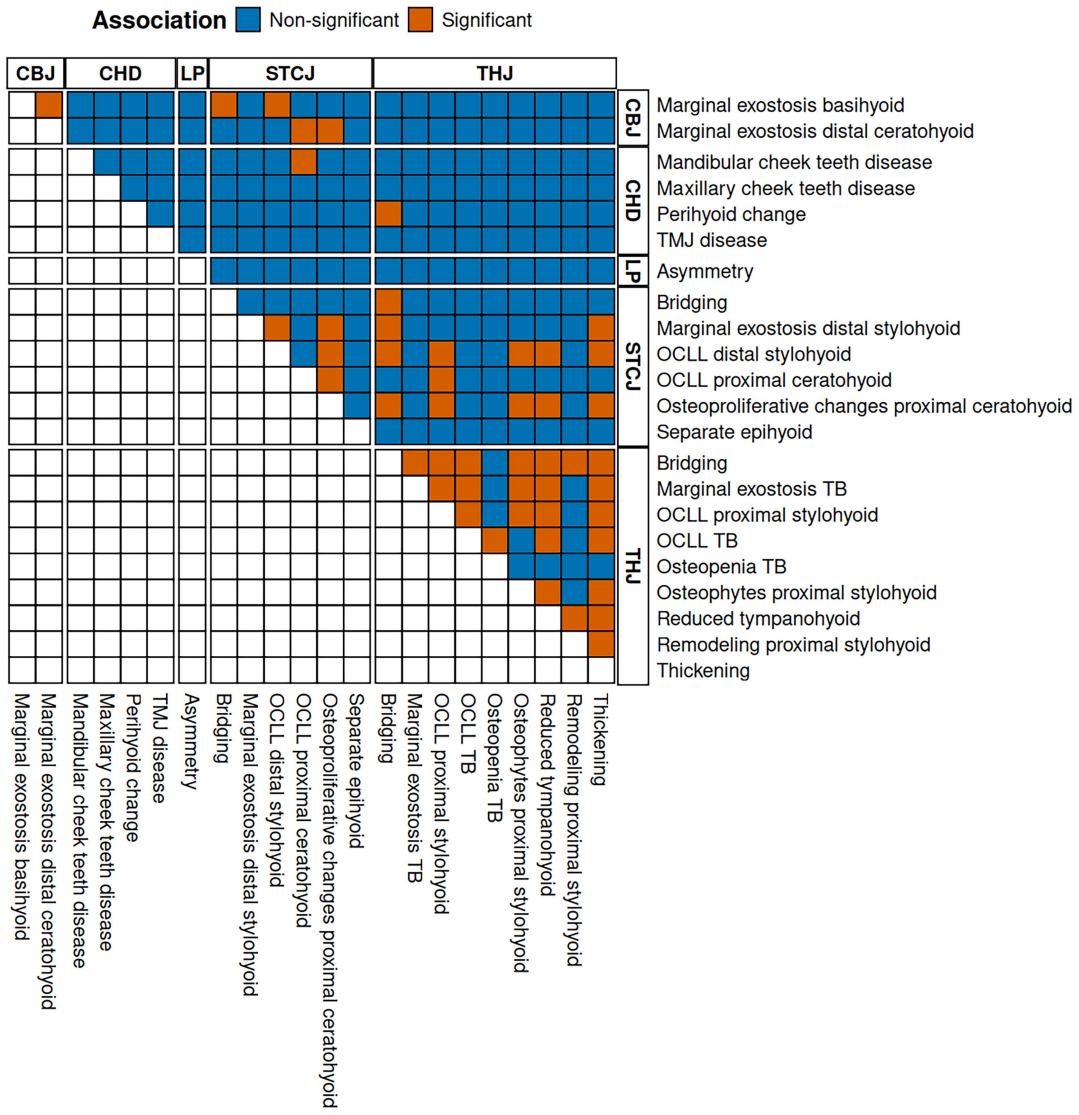
Illustration of the association tests with dichotomized adjusted *p*-value at a significance cut-off of 5%. Orange tiles show a significant association between the parameters listed on the right-hand and the upper side. The letters at the top and right indicate the category of change. (CBJ) ceratobasihyoid joint, (CHD) concurrent head disease, (LP) lingual process, (OCLL) osseous cyst-like lesions, (STCJ) styloceratohyoid/epiceratohyoid joint, (TB) temporal bone, (THJ) temporohyoid joint.

The results of the association tests are listed in Supplementary Table S2 and visualized in Figure 4. Most THJ parameters (8/9) were significantly associated with other THJ parameters, mostly with bridging, thickening, or reduced tympanohyoid. Similarly, 3/6 CT findings in the STCJ were significantly associated with other pathologies of the STCJ. In the CBJ, both parameters were found to be associated with each other.

Table 3 lists three CT signs in which a bone parameter, such as OCLL of the distal stylohyoid, was associated with the same (OCLL proximal stylohyoid) or a different parameter (osteophytes proximal stylohyoid) of the same bone.

**Table 3:** Same or different parameters of the same bone associated with each other.

Evaluated parameter	Associated parameter
Marginal exostosis distal ceratohyoid	OCLL proximal ceratohyoid Osteoproliferative changes proximal ceratohyoid
Osteophytes proximal stylohyoid	OCLL distal stylohyoid
OCLL proximal stylohyoid	OCLL distal stylohyoid

OCLL, Osseous cyst-like lesion.

In almost all cases, diseases in the STCJ were significantly associated with diseases of the THJ. These CT signs are listed in Table 4 and include the same CT signs as bridging of the styloceratohyoid joint and bridging of the THJ, as well as different signs such as osteophytes of the proximal stylohyoid and osteoproliferative changes of the proximal ceratohyoid.

**Table 4:** Abnormalities of the styloceratohyoid/epiceratohyoid joint associated with changes of the temporohyoid joint.

STCJ-changes	THJ-changes
Marginal exostosis distal stylohyoid	Thickening THJ Bridging THJ
OCLL distal stylohyoid	Bridging THJ Reduced tympanohyoid Thickening THJ
Bridging styloceratohyoid joint	Bridging THJ
OCLL proximal ceratohyoid	OCLL proximal stylohyoid
Osteoproliferative changes proximal ceratohyoid	Bridging THJ OCLL proximal stylohyoid Osteophytes proximal stylohyoid Reduced tympanohyoid Thickening THJ

OCLL, Osseous cyst-like lesion; STCJ, Styloceratohyoid/epiceratohyoid joint; THJ, Temporohyoid joint.

CBJ CT signs of disease, such as marginal exostosis of the basihyoid, were significantly associated with STCJ diseases, such as bridging of the STCJ and OCLL of the distal stylohyoid.

Diseases of the mandibular cheek teeth were significantly associated with OCLL of the proximal ceratohyoid. Perihyoid changes were associated with bridging of the THJ.

### 3.4 Effect of age, weight, and use of the horse on the occurrence of changes in the evaluated parameters

Thirteen variables were used to assess the effect of age, use, and weight of the horse. A positive effect of age was demonstrated on the following THJ parameters: marginal exostosis of the TB, OCLL of the TB, and proximal stylohyoid, thickening of the THJ, reduced tympanohyoid, and osteophytes of the proximal stylohyoid. Furthermore, a positive effect of age was detected on these STCJ signs: marginal exostosis of the distal stylohyoid and osteoproliferative changes at the proximal ceratohyoid (Supplementary Table S3). Age did not have an effect on the development of cheek teeth abnormalities nor on TMJ diseases.

The use and weight of the horses did not affect the occurrence of any of the CT signs evaluated (Supplementary Tables S4, S5).

## 4 Discussion

Documented cases of hyoid bone pathologies are described in the literature and have been known to result in exercise intolerance, equine riding problems, and dysphagia. The method of choice for examining the hyoid bone in horses is computed tomography. Therefore, in this study, we described and quantified the hyoid bone pathologies occurring in a subset of horses that, for various reasons, had undergone CT scans of the head. The prevalence of disease was found to be highest at the THJ, followed by the STCJ. In contrast, the CBJ was identified to be the joint with the lowest prevalence of disease. Significant associations were observed among CT signs in the examined joints of the hyoid apparatus. Age influenced the presence of THJ and STCJ abnormalities.

Several studies on THO describe CT signs of disease in the THJ, such as proliferation of the TB and stylohyoid, and fractures of these bones (6, 7, 26). Stylohyoid proliferation was found in 16/16 horses (7) and 2/2 horses (6), TB fractures in 4/16 (7) and 16/39 horses (15) and stylohyoid fractures in 4/16 horses with THO (7). Osteophytes at the joint margins (16, 18) and remodeling of the proximal stylohyoid (16, 18) have been seen in horses with age-related degeneration of the THJ. In the present study, the most common CT signs of disease of the hyoid apparatus occurred at the THJ: 52% of the sides examined showed a marginal exostosis of the TB, and 44% showed osteophytes of the proximal stylohyoid. Remodeling of the proximal stylohyoid, as seen in THO, occurred in only 4% (13/322) of the sites examined in our population. The relatively high percentage of 52% of the sides showing marginal exostosis of the TB could be explained by age-related primary osteoarthritis, as described by Naylor et al. (18) as lipping of the TB. Additionally, the styloid process sheath, a bony sheath of the TB surrounding the styloid process; the tympanohyoid, and the caudodorsal part of the stylohyoid (27, 28), were interpreted as marginal exostosis of the TB in this study. A significant association with age has also been described for the styloid process sheath size (29). In summary, the findings of this study revealed that age-related degenerative disease at the THJ predominated in the mixed study population.

The STCJ is the synovial joint between the stylohyoid and ceratohyoid, or, if the epihyoid is not fused to the stylohyoid, the joint between the epihyoid and ceratohyoid (5). A separate epihyoid was found in 33% (66/200) (5) of horses, and 38.6% (112/290) (17) of horses in other studies, and was presumed to be an ossification center of the stylohyoid (5). Thickening of the ceratohyoid and a bony proliferation of the joint have been reported in horses with THO (7, 19). In this study, a separate epihyoid was present in 38.7% (127/328) of the examined sides. In addition, marginal exostosis of the distal stylohyoid was found in 29% (95/328) of the sides examined, and osteoproliferative changes of the proximal ceratohyoid in 16.5% (54/328) of the sides examined. Age influenced the frequency of the latter two parameters, which enabled us to visualize that, in addition to abnormalities of the STCJ associated with THO, degenerative disease of this joint also occurs regularly.

The CBJ has a spacious joint space with a smaller craniomedial and a larger caudolateral recess (5). Disease of this joint is not reported in the literature. In one case of hyoid malformation, the joint was reported to be widened on the side affected by the malformation (2). In a recent report on hyoid malformation, the joint appeared to be fused (21). In another case report, a fracture of the horizontal part of the basihyoid was described, but without involvement of the CBJ (4). The most common disease observed in this joint, as reported in this study, was a marginal exostosis of the basihyoid in 2.7% (9/328) of the examined sides. The other two CT signs of this joint were rare, with 1.5% (5/328) for the basihyoid OCLL and 1.2% (4/328) for the distal ceratohyoid OCLL. CT signs of the CBJ were associated with those of the STCJ, and the CT findings in this joint were related to the CT signs of the THJ. It is therefore proposed that CBJ abnormalities, despite their low prevalence in the CBJ and the fact that they represent the least common joint disease of the hyoid apparatus, may, in fact, also be observed in cases of THJ disease. Given its accessibility via ultrasonography, more frequent evaluation of the CBJ in horses with ridden performance issues may facilitate earlier detection and increased reporting of joint disease.

The basihyoid consists of: the LP, which is embedded in the body of the tongue; the corpus or horizontal part, which lies in the root of the tongue; and the two thyrohyoid processes, which connect the hyoid apparatus to the thyroid cartilage of the larynx. The CT appearance of the LP varies with age (5): Under 1.5 years of age, it is about half the size of an adult horse (5). Between 1.5 and 3 years, a separate ossification center is visible for the tip of the LP (5). We did not examine developmental forms in this study and therefore excluded all horses under 3 years of age. There are reports in the literature of an LP fracture with refusal to take the bit and collect the neck when ridden, as well as headshaking (3); as well as a report of a basihyoid fracture with an inability to eat and drink, and showing resentment toward any manipulation of the tongue (4). In this study, we found an LP fracture in 1/160 (0.6%) of the horses and an LP asymmetry in 9/152 (5.9%) of the horses. Although diseases of the LP are quite rare, when they do occur, they can affect the ability to eat and drink, as well as their ridden performance. In this study, CT scans were performed on horses with LP disease to detect more serious problems, such as mandibular fractures or space-occupying masses in the head region. This leads us to conclude that both LP asymmetry and fracture can occur without severe clinical symptoms; however, as described in the literature, they need to be considered when other pathologies cannot be detected.

One proposed aetiopathogenesis of THO is progressive otitis media, partly as an extension of otitis externa, which appears to spread ventrally and which causes osteitis of the tympanic bulla, of the TB, and of the stylohyoid (30, 31). A recent study found no association between imaging evidence of otitis media or externa and THO (16). Postmortem CT scans were excluded from the assessment of signs of disease of the external and/or middle ear in this study because there was a greater amount of fluid in the tympanic bulla and external ear canal on postmortem CT scans compared to CT scans of live animals. Signs of external/middle ear disease were found in 6/273 sides of horses examined in the present study. Of these, 4 (67%) showed no radiological signs of THO, and 2 (33%) had radiological signs of THO. In this study, a small number of horses exhibited signs of external/middle ear disease, while many horses had THJ disease. Nevertheless, the present study did not specifically examine CT signs of THO per se; consequently, it was not possible to evaluate a correlation between THO CT signs and signs of external or middle ear disease.

OCLLs are radiolucent areas of bone, with a sclerotic rim next to the weight-bearing part of the articular surface (32–35). Possible underlying pathomechanisms of OCLLs include: developmental disorders, trauma, or focal sepsis (36). They may be associated with clinical problems or represent an incidental radiographic finding (36). In this study, the presence of OCLLs at all joints of the hyoid apparatus was investigated. They were present in all joints and bones with varying frequencies. As with all CT signs, OCLLs were most commonly found at the THJ, with 44/323 (14%) of the sides examined showing OCLLs at the proximal stylohyoid, and 34/313 (11%) of the sides examined showing OCLLs at the TB. At the STCJ, OCLLs were detected on 24/328 (10%) of the sides examined on the distal stylohyoid and 25/328 (8%) of the sides examined on the proximal ceratohyoid. In 4/328 (1%) of the CBJ, OCLLs appeared on the distal ceratohyoid, and in 5/328 (2%) of the sides examined, OCLLs appeared on the basihyoid. 10/25 (40%) OCLLs of the proximal ceratohyoid occurred together with diseases of the mandibular cheek teeth, on the same side. The statistical analysis revealed a significant association between these two parameters. Of the sides where diseases co-occurred, there were mandibular fractures on two sides and a squamous cell carcinoma on two other sides. This would explain the observed association between these two parameters. In the case of mandibular fractures, the OCLLs of the proximal ceratohyoid could have been caused by the trauma itself. In the case of the squamous cell carcinoma of the mandible, the OCLLs could have been caused by focal sepsis.

The study by Naylor et al. (18) reported on the age-related degeneration of the THJ. Subsequent studies have identified associations between age-related CT signs in the THJ (23, 29) and alterations in the remaining hyoid apparatus (23). However, no correlation was found between clinical symptoms and THJ grading (16), nor was there an association between CT findings of the THJ and headshaking (17). Additionally, Thoroughbreds and Arabians have been suggested to exhibit a predisposition to more pronounced THJ remodeling, whereas the Cob breed may possess a protective effect (16). In the present study, age was found to significantly influence six parameters of the THJ and two parameters of the STCJ. The effect of breed was not evaluated due to multicollinearity with other variables. However, the potential influence of use and weight was analyzed, and no significant effects of these factors on the evaluated CT signs were identified. These findings further support the role of age in the development of THO and reveal degenerative disease in the STCJ. Conversely, use and weight appear to have no significant impact on the progression of changes to the hyoid apparatus.

In conclusion, this study presents a detailed collection of the computed tomographic CT signs of disease in all joints of the hyoid apparatus. The most common diseases were seen in the THJ. Disease in the CBJ was associated with those in the STCJ, which, in turn, were associated with disease in the THJ. Age had an effect on six THJ and two STCJ CT signs. Use and weight, however, did not have an effect on the development of CT signs of disease of the hyoid. Given the frequencies of specific CT signs, particularly in the STCJ and the CBJ, these findings may assist clinicians distinguish between normal age-related degeneration and potentially clinically significant CT diseases. In future studies, the clinical relevance of the hyoid joint disease should be analyzed, possibly with the refinement or establishment of tests to examine the different joints of the hyoid bone. Additionally, the effect of the hyoid joints on ridden performance should be evaluated.

## Data Availability

The original contributions presented in the study are included in the article/Supplementary material, further inquiries can be directed to the corresponding author.

## Author’s note

Parts of the results included in this paper have been presented at the 21st IGFP-Conference, 15-16 March 2024 in Niedernhausen, Germany and at the 31st Veterinary Dental Forum, 31 May–1 June 2024 in Nantes, France.
